# Considerations of Timing Post-ovariectomy in Mice and Rats in Studying Anxiety- and Depression-Like Behaviors Associated With Surgical Menopause in Women

**DOI:** 10.3389/fnbeh.2022.829274

**Published:** 2022-03-04

**Authors:** Juan Francisco Rodríguez-Landa

**Affiliations:** Laboratorio de Neurofarmacología, Instituto de Neuroetología, Facultad de Química Farmacéutica Biológica, Universidad Veracruzana, Xalapa, Mexico

**Keywords:** anxiety, depression, oophorectomy, psychopharmacology, surgical menopause model

## Introduction

Menopause is a clinical term that indicates the end of the reproductive period of women that occurs naturally; however, it can be induced by bilateral oophorectomy, which is commonly referred to as surgical menopause. Both types of menopause in women are characterized by low plasma levels and brain concentrations of estradiol and progesterone, and a marked increase in follicle-stimulating hormone (FSH) levels. This likely affects several brain neurotransmitter systems and some peripheral physiological processes, impacting the quality of life of women (Monteleone et al., 2018; Giannini et al., 2021). In addition, the long-term absence of steroid hormones is associated with physiological changes that predispose women to genitourinary, cardiovascular, osseous, and mood disorders (Giannini et al., 2021). While such changes occur gradually and require a long time to stabilize in natural menopause, they are established in a shorter time in surgical menopause, thereby influencing the severity of symptoms in comparison to natural menopause (Rodríguez-Landa et al., 2015; Kingsberg et al., 2020; Giannini et al., 2021).

In preclinical research, ovariectomy performed on mice and rats is used as a surgical menopausal model to study the effects of permanent reduction in the levels of steroid hormones. This model has been used for studying the effects of surgical menopause on the central nervous system, which is responsible for cognitive deterioration, irritability, anxiety- and depression-like behaviors (Zakaria et al., 2019; Georgieva et al., 2021). In this way, the present article discusses the influence of time post-ovariectomy on hormonal, neurochemical, and neuroanatomical changes in rodents and its relation with anxiety- and depression-like behaviors that have been reported among women subjected to bilateral oophorectomy. This would help in enhancing the understanding of behavioral changes associated with surgical menopause and help evaluate potential pharmacological strategies to ameliorate the negative effects produced by long-term ovariectomy.

## Menopause, Anxiety, and Depression Symptoms

Throughout the reproductive life of the human female, significant changes in the peripheral and central concentrations of estradiol and progesterone have been reported. These hormonal changes interfere with their physiological, endocrine, and neurochemical processes, and often have negative implications on their quality of life (Kingsberg et al., 2020). Reduced concentrations of estradiol and progesterone during the premenstrual, postpartum, and menopausal periods predispose women to anxiety and depressive symptoms (Albert et al., 2015). Generally, natural menopause occurs at 45–55 years of age and indicates the end of the reproductive period in women. However, surgical menopause, which is artificially induced by bilateral oophorectomy (Rodríguez-Landa et al., 2015), also exists as an option. Oophorectomy is recommended to women for averting the potential development of estrogen-dependent ovarian/breast cancer or to reduce pain from endometriosis (Kingsberg et al., 2020). Both types of menopause are known to predispose women to neuropsychiatric disorders (Georgieva et al., 2021), which is attributed to low concentrations of estradiol, progesterone, and their reduced metabolites such as allopregnanolone, among others (Zsakai et al., 2016).

Clinical studies have shown that women who undergo bilateral oophorectomy develop physiological and psychological changes similar to those that occur with natural menopause. One report suggest that women can immediately develop symptoms of severe anxiety that impair their quality of life (Chung-Park, 2006). The emotional and affective symptoms associated with surgical menopause vary among women; however, it has been reported that performing bilateral oophorectomy before natural menopause is associated with the development of anxiety and depression disorders and an increased risk in cognitive impairment, compared with women who experience natural menopause (Rocca et al., 2018; Olmos-Vázquez et al., 2020). Rocca et al. (2018) reported an increased long-term risk of depression and anxiety symptoms in women who underwent bilateral oophorectomy, which was principally associated with estrogen deficiency. Interestingly, the incidence of anxiety symptoms in bilateral oophorectomized women was higher in the long-term as compared to that of non-oophorectomized women, which exponentially increased as time post-oophorectomy passed, even, 10, 20, and 30 years after the removal of both ovaries. As such, preclinical and clinical studies are required to understand the neurobiological bases of the disorders associated with surgical menopause in the long-term, thus enabling the evaluation of new therapeutic strategies to ameliorate symptoms and improve the quality of life of this particular group of women. In this case, neuroendocrine changes produced by long-term ovariectomy should be considered when this surgical procedure is used for evaluating the disorders associated with surgical menopause and their potential treatments.

## Effects of Long-Term Ovariectomy on Anxiety- and Depression-Like Behavior

It is important to emphasize that multiple changes in the levels of diverse hormones and other substances (e.g., estradiol, progesterone, testosterone, cortisol, prolactin, insulin, glucose, and lipids) occur during natural or surgical menopause in the human female (Huerta et al., 1995; Kingsberg et al., 2020), which contribute to multiple symptoms associated with menopause. However, the most significant changes in natural or surgical menopause among women include a decrease in early cycle inhibin B and anti-Müllerian hormone levels. The decline in inhibin B produces an increase in FSH levels, which regulate the production of estradiol. Therefore, in menopausal women, FSH levels are markedly raised, and estradiol levels are significantly lowered (Burger et al., 2007; Kingsberg et al., 2020); these are associated with characteristic symptoms during menopause. Nonetheless, these hormonal changes are different between natural and surgical menopause. These changes occur gradually in natural menopause, while in surgical menopause, they occur in a shorter time.

In preclinical research, ovariectomy is a surgical procedure that involves the removal of one or both ovaries in females, which in the medium- and long-term significantly reduces plasma and brain concentrations of steroid hormones and other molecules that negatively impact brain function (Alagwu and Nneli, 2005). Interestingly, in ovariectomized mice and rats, similar hormonal changes to those of the human female occur. The FSH levels are increased as more time passes following ovariectomy, which is higher at 4 weeks than at 1–2 weeks (Moiety et al., 2015). Contrarily, progesterone and estradiol levels are undetectable at 3 months and 15 months post-ovariectomy, respectively, and they are lower at 4 weeks, than they are at 1–2 weeks post-ovariectomy (de Chaves et al., 2009; Moiety et al., 2015).

It has been reported that apparition of anxiety- and depression-like behavior associated with ovariectomy depends on the time elapsed after the procedure. For instance, the incidence of anxiety-like behavior was higher in rats at 12 weeks than at 3 weeks post-ovariectomy (Picazo et al., 2006); however, a limitation of this study was that these results were not compared with those of non-ovariectomized rats. Interestingly, 1 week post-ovariectomy did not produce significant changes on anxiety-like behavior with respect to non-ovariectomized rats; however, 3 weeks post-ovariectomy, a high incidence of anxiety-like behavior was detected in comparison with that of rats in the proestrus-estrus phase. This effect was similarly detected in rats at 6, 9, 12, and 15 weeks post-ovariectomy (Puga-Olguín et al., 2019), which coincides with estradiol and progesterone levels reduction. It was reported that rats at 12 weeks post-ovariectomy were more responsive to diazepam, an anxiolytic drug, than rats at 3 weeks post-ovariectomy (Picazo et al., 2006). This emphasizes the importance of considering the time post-ovariectomy in the evaluation of anxiety-like behavior and the effect of anxiolytic drugs.

Moreover, in the forced swim test an increase in the time of immobility was detected 2 weeks post-ovariectomy (Fedotova et al., 2016). Contrarily, Puga-Olguín et al. (2019) did not detect depression-like behaviors (increased time of immobility) in rats at 1 and 3 weeks post-ovariectomy, with respect to non-ovariectomized rats. Nonetheless, rats from 6 weeks post-ovariectomy significantly increased the incidence of depression-like behavior, with respect to rats in proestrus-estrus and metestrus-diestrus phases, and this effect was similar in rats at 9, 12, and 15 weeks post-ovariectomy.

As mentioned above, the time post-ovariectomy in mice and rats plays a significant role in the expression of anxiety- and depression-like behavior, which can be partially explained by the hormonal, neurochemical, and neuroanatomical changes associated with ovariectomy in the long term. It is noteworthy that all these changes are simply an overview of how hormones affect the brain in the context of anxiety and depression symptoms, but we cannot disregard the other physiological changes that may influence symptoms related to menopause.

## Effects of Long-Term Ovariectomy on Follicle-Stimulating Hormone and Steroid Hormones in Rats and Mice

Early studies have explored endocrine changes associated with ovariectomy in rats and mice. Particularly in rats, 2 weeks post-ovariectomy, the plasma concentrations of estradiol and progesterone did not differ significantly from the basal conditions (Ratka and Simpkins, 1990). However, 3 weeks post-ovariectomy, there was a significant reduction in estradiol and progesterone levels and an increase in luteinizing hormone and FSH levels in plasma (Wise and Ratner, 1980). To note, the reduced concentration of estradiol after 2 weeks post-ovariectomy remained until 7 weeks when the study ended (Li et al., 2014). Six weeks post-ovariectomy, a significant reduction in the plasma levels of progesterone, estradiol, and testosterone was detected in rats (Alagwu and Nneli, 2005).

On the other hand, Moiety et al. (2015) evaluated the effects at 1 and 4 weeks after hysterectomy and unilateral and bilateral ovariectomy on FSH and estradiol levels in rats. With respect to the baseline, FSH levels increased approximately to 26.19 and 73.81%, after 1 and 4 weeks post-hysterectomy, respectively. One week post unilateral ovariectomy, FSH levels increased approximately to 44.68%, while after 4 weeks FSH increased to ~80.85% with respect to the baseline. Interestingly, a more significant effect on FSH levels was observed when bilateral ovariectomy was performed. In this case, 1 week post bilateral ovariectomy FSH levels significantly increased to approximately 91.11% vs. the baseline, but more significant effects were detected after 4 weeks, increasing FSH approximately to 222.22% with respect to the baseline. As for the concentration of estradiol at 1 week post hysterectomy, a slight reduction of ~5.21% was noted, while 4 weeks after, estradiol levels were reduced to ~16.94%, compared with the baseline. One week after the unilateral ovariectomy, estradiol was reduced to 7.26%, while 4 weeks after it was reduced to ~29.19%, with respect to the baseline. Interestingly, when bilateral ovariectomy was performed, the concentration of estradiol was reduced to ~21.45% of the baseline. However, estradiol was further reduced to 60.81% 4 weeks post bilateral ovariectomy with respect to the baseline. Importantly, the greater effects on FSH and estradiol were produced after 4 weeks of bilateral ovariectomy, followed by unilateral ovariectomy and hysterectomy. These results are consistent with those of previous studies that found a significant reduction in plasma concentrations of estradiol and testosterone at 4 weeks post-ovariectomy and was undetectable at 24 weeks post-ovariectomy (Zhao et al., 2005). The same effect was detected in rats at 3 months and 15 months post-ovariectomy (de Chaves et al., 2009).

Despite few studies on endocrine changes occurring in ovariectomized mice, it has been reported that after 4 days and 1 week post-ovariectomy, the concentrations of FSH are significantly increased with respect to intact cycling mice (Bronson, 1976; Chandrashekar and Bartke, 1996; Xu et al., 2000), but greater effects are detected after 2–4 weeks post-ovariectomy (Rodin et al., 1990), as it occurs in ovariectomized rats. Accordingly, after 2–14 weeks post-ovariectomy in mice, serum estradiol levels were significantly decreased by approximately 31% with respect to the sham-operated mice, which was accompanied by a significantly increased concentration of FSH (Park et al., 2014; Lee et al., 2020; Canuas-Landero et al., 2021). These data clearly show the importance of considering and standardizing the post-ovariectomy time frame to identify behavioral and hormonal changes associated with this surgical manipulation, thus avoiding erroneous interpretation of results and compare results from different laboratories.

## Neuroanatomical and Neurochemical Changes Associated with Long-Term Ovariectomy: Effects on Anxiety and Depression-Like Behavior

The lower concentrations of progesterone and estrogen that occur during surgical or natural menopause produce an imbalance in neurochemical brain communication that subsequently affects neurotransmitter pathways (Smith et al., 1998; Rodríguez-Landa et al., 2015; García-Ríos et al., 2017; Kingsberg et al., 2020), wherein reduced concentrations of dopamine, serotonin, androstenedione, testosterone estradiol, GABA, β-endorphin, and allopregnanolone, and increased concentrations of noradrenaline and cortisol, among others, have been reported (Monteleone et al., 2018). All these changes produce neuroanatomical modifications in brain structures involved in anxiety and depression such as the raphe nucleus, hippocampus, and cerebral cortex (Monteleone et al., 2018).

In experimental research, bilateral ovariectomy in rats has also been used to evaluate the effects of the post-ovariectomy time frame on anxiety- and depression-like behavior, as well as the effect of anxiolytic and antidepressant compounds under these experimental conditions (Table 1). However, different post-ovariectomy time frames in mice and rats have been used, which can hinder the comparison of the results of different studies. Therefore, it is important to establish the different changes associated with the time of post-ovariectomy in rodents.

**Table 1:** Effects of timing post-ovariectomy and some pharmacological treatments on anxiety- and depression-like behaviors in mice and rats.

**Effects of ovariectomy and anxiolytic drugs on anxiety-like behavior**				
**Subjects/Weeks post-OVX**	**OVX effect/Test**	**Treatment**	**Treatment effect** ^ 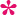 ^	**References**
NMRI mice/1	ne/EPM	–	–	Galeeva and Tuohimaa, 2001
Wistar rats/1	ne/EPM	–	–	Puga-Olguín et al., 2019
Sprague-Dawley rats/2	ne/OFT	–	–	Hiroi and Neumaier, 2006
C57BL/6 mice/2	ne/CUS+EPM	–	–	Lagunas et al., 2010
Wistar rats/3	ne/EPM	–	–	Marcondes et al., 2001
Wistar Rats/8	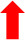 /EPM	–	–	Dornellas et al., 2018
Wistar rats/3,6,9,12,15	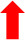 /EPM	–	–	Puga-Olguín et al., 2019
Sprague-Dawley rats/4	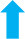 /EPM	–	–	Zoladz et al., 2019
C57BL/6 mice/16	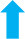 /CUS+EPM	–	–	Lagunas et al., 2010
Wistar rats/12	ne/EPM	–	–	de Chaves et al., 2009
Wistar rats/60	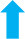 /EPM	–	–	de Chaves et al., 2009
Long-Evans rats/1	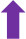 /DBT	4 mg/kg P 10 μg/rat E2	P reduces anxiety-like behavior, E2 without effect	Llaneza and Frye, 2009
Sprague-Dawley rats/2	ne/EPM	6 μM/rat Allo	ne	Laconi et al., 2001
Sprague-Dawley rats/2	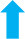 /EPM	25 mg/kg P 10 μg/kg E2B	ne	Díaz-Véliz et al., 1997
Wistar rats/3	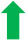 /LDB	1 mg/kg DZ	Reduces anxiety-like behavior	Zuluaga et al., 2005
Wistar rats/3, 12	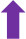 /DBT	0.5, 1, and 2 mg/kg DZ 0.5 and 0.50 mg/kg 8-OH-DPAT	Both treatments reduce anxiety-like behavior	Picazo et al., 2006
Wistar rats/4	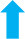 /EPM	10 μg/kg E2 10 mg/kg Flx	E2 reduces anxiety-like behavior, Flx without effect	Charoenphandhu et al., 2011
Wistar rats/4	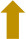 /OFT	150 μg/rat/week E2	Reduces anxiety-like behavior	Diz-Chaves et al., 2012
Wistar rats/8	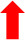 /EPM	0.09 mg/kg E2	Reduces anxiety-like behavior	Puga-Olguín et al., 2019
Wistar rats/12	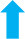 /EPM	0.5, 1, 2, and 4 mg/kg Chry	Only 1, 2, and 4 mg/kg Chry reduce anxiety-like behavior	Rodríguez-Landa et al., 2019
Wistar rats/12	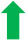 /LDB	0.25, 0.5, and 1 mg/kg Gen	Reduces anxiety-like behavior	Rodríguez-Landa et al., 2009
Wistar rats/12	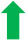 /LDB	2 mg/kg Dz	Reduces anxiety-like behavior	Rodríguez-Landa et al., 2019
Wistar rats/12	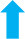 /EPM	0.45, 0.09, and 0.18 mg/kg E2 and Gen	Only 0.09 and 0.18 mg/kg reduce anxiety-like behavior	Rodríguez-Landa et al., 2017
**Effects of ovariectomy and antidepressant substances on depression-like behavior**				
**Animal/Weeks post-OVX**	**OVX effect/Test**	**Treatment**	**Treatment effect** ^** 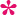 **^	**References**
C57BL/6 mice/2	ne/CUS+FST	–	–	Lagunas et al., 2010
C57BL/6 mice/2	ne/FST 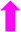 /TST	–	–	Carrier et al., 2015
Wistar rats/1, 3	ne/FST	–	–	Puga-Olguín et al., 2019
Wistar rats/8	ne/FST	–	–	Dornellas et al., 2018
Wistar rats/12, 60	ne/FST	–	–	de Chaves et al., 2009
Wistar rats/6, 9, 12, 15	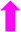 /FST	–	–	Puga-Olguín et al., 2019
C57BL/6 mice/16	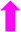 /CUMS+FST	–	–	Lagunas et al., 2010
C57BL/6 mice /1-2	ne/TST	10 mg/kg P	Reduces depression-like behavior	Frye, 2011
Wistar rats/2	ne/FST	0.8, 1.6, and 3.0 mg/kg P	Reduces depression-like behavior	Martínez-Mota et al., 1999
Wistar rats/2	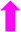 /FST	0.3, 1, and 3 μg/rat E2 and 0.6 mg/rat Map	Reduces depression-like behavior	Okada et al., 1997
Wistar rats/2	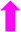 /FST	1 mg/kg P, Allo, Chry	Reduces depression-like behavior	Cueto-Escobedo et al., 2020
Wistar rats/7	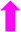 /FST	150 μg/rat/week E2	Reduces depression-like behavior	Diz-Chaves et al., 2012
Wistar rats/8	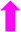 /FST	0.09 mg/kg E2	Reduces depression-like behavior	Puga-Olguín et al., 2019
Wistar rats/8	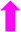 /FST	0.5, 1, and 2 mg/kg P	Only 1 and 2 mg/kg P reduce depression-like behavior	Rodríguez-Landa et al., 2020
ICR mice/8	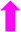 /FST /TST	1 μg/kg E2 and 100 mg/kg Pue	Both treatments reduce depression-like behavior	Tantipongpiradet et al., 2019
Wistar rats/8	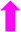 /FST	5 mg/kg P	Reduces depression-like behavior	Rodríguez-Landa et al., 2020
Wistar rats/9	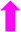 /FST	25 μg/day/6 weeks E2	Reduces depression-like behavior	Khayum et al., 2020
Wistar rats/12	ne/FST	0.25 mg/rat E2	ne	Boldarine et al., 2019

*Behavioral tests: CUS, chronic unpredictable stress; OFT, open field test; DBT, defensive burying test; LDB, light/dark box; FST, forced swim test; TST, tail suspension test. Evaluated substances: E2, estradiol; E2B, estradiol benzoate; P, progesterone; Allo, neurosteroid allopregnanolone; Flx, fluoxetine; Map, maprotiline; Dz, diazepam; Chry, flavonoid chrysin; Gen, isoflavone genistein; Pue, isoflavone puerarin. Effects of timing post-ovariectomy: ne, no effects on anxiety- or depression-like behaviors; 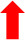, OVX increase anxiety-like behavior by decreasing time in open arms and increasing anxiety index; 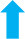, OVX increase anxiety-like behavior by decreasing time in open arms; 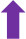, OVX increase anxiety-like behavior by increasing time spent burying; 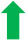, OVX increase anxiety-like behavior by decreasing time spent in light compartment; 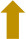, OVX increase anxiety-like behavior in aged rats by decreasing time in central areas; 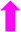, OVX increase depression-like behavior by increasing total time of immobility; ^
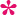
^Anxiety- and depression-like behaviors are reduced when treatment produce the contrary effect on the variable described in the second column of the table (indicated by the different arrow colors). OVX, ovariectomy*.

Ovarian hormones, estradiol, and progesterone, regulate several neurochemical processes in brain structures involved in the physiopathology of anxiety and depression, such as the prefrontal cortex, hypothalamus, amygdala, septal nucleus, and hippocampus (Giannini et al., 2021). Studies among laboratory animals and humans have reported that progesterone and its reduced metabolite allopregnanolone target the GABA_A_ receptor and facilitate the activation of the GABAergic system, which can modulate dopaminergic and serotonergic pathways (Chen et al., 2021), producing anxiolytic- and antidepressant-like effects (Fernández-Guasti and Picazo, 1995; Estrada-Camarena et al., 2002; Frye and Walf, 2002; Rodríguez-Landa et al., 2007). Estrogen appears to mediate anxiety- and depression-like behavior by stimulating tryptophan hydroxylase, the rate-limiting enzyme in the synthesis of serotonin (Bethea et al., 2000; Giannini et al., 2021). In rats, estrogen also mediates the regulation of the 5-hydroxytryptamine-1A (5-HT_1A_) receptor expression in the dorsal raphe nucleus. Furthermore, it increases the density of postsynaptic 5-HT_2_ receptors in the forebrain (Sumner et al., 1999), thereby affecting the receptors involved in the neurobiology of depressive disorders and the mechanisms of action of antidepressant drugs (García-Ríos et al., 2017).

Interestingly, at 1 week post-ovariectomy in rats, there was a reduction in the activation of glutamatergic receptors in the basolateral amygdala (De Jesús-Burgos et al., 2012), a structure involved in the regulation of anxiety. At 1–2 weeks post-ovariectomy, a critical reduction of dendritic spines and synaptophysin density occurred in the pyramidal neurons of the CA1 layer of the hippocampus in rats (Velázquez-Zamora et al., 2012), negatively impacting neuronal communication and the regulation of emotional and cognitive processes. This reduction in dendritic spine density in CA1 also occurs in rats at 3 weeks post-ovariectomy (McLaughlin et al., 2008). It is important to note that neurochemical changes in the brain are dependent on the post-ovariectomy time frame. In this way, after 4 weeks post-ovariectomy, genomic changes occur in the brain structures involved in the neurobiology of anxiety and depression. As studied in rats, the mRNA expression of the α-2 and α-3 subunits of the amygdala GABA_A_ receptor (De Jesús-Burgos et al., 2012) was reduced, along with tryptophan hydroxylase mRNA, and serotonergic transporter mRNA of the dorsal raphe nucleus (Charoenphandhu et al., 2011). In addition, the concentration of serotonin in the hippocampus and nucleus accumbens (Pandaranandaka et al., 2009) is reduced. Additionally, other neurochemical and molecular changes appear post-ovariectomy. It is noteworthy that all these changes are reported from 4 weeks post-ovariectomy, but it is unknown whether these changes occur earlier than 4 weeks post-ovariectomy, which remains to be explored.

Five weeks after ovariectomy in mice, there is a reduced thickness of the CA1 layer in the hippocampus and the cerebral prefrontal cortex (Xu and Zhang, 2006). While 6 weeks after ovariectomy in rats, there is a reduction in the dopamine concentration in the central nucleus of the amygdala (Izumo et al., 2012) and a significant reduction in the expression of Fos protein immunoreactivity in the dorsal, intermediate, and ventral areas of the lateral septum. However, this was negatively correlated with anxiety- and depression-like behavior (Puga-Olguín et al., 2019). Interestingly, at 8 and 9 weeks post-ovariectomy, a reduced expression of estrogen receptors (ER), αER and βER mRNA was detected in the hippocampus and cerebral cortex along with a reduced density of dendritic spines in the cerebral cortex of rats (Jin et al., 2005).

All these data clearly show that ovariectomy in mice and rats produces significant neuroanatomical and neurochemical changes in brain structures involved in the physiopathology of anxiety and depression, in which anxiolytic and antidepressant drugs play a contributory role, similar to women experiencing early surgical menopause (Kingsberg et al., 2020; Georgieva et al., 2021). Although few studies have correlated neurochemical and neuroanatomical changes that occur in ovariectomy with anxiety- and depression-like behaviors, these results support that long-term ovariectomy can be a useful tool to evaluate the neurobiological substrates that underlie anxiety- and depression-like behaviors associated with a reduced concentration of ovarian hormones induced by surgical menopause. Further, consideration of these factors will aid in studying the effect of new compounds with potential anxiolytic and antidepressant activity in women whose ovaries are removed.

## Evaluation of Anxiety- and Depression-Like Behavior Associated with Long Term Ovariectomy: Effects of Anxiolytic and Antidepressant Drugs

Long-term ovariectomy in mice and rats increases the incidence of anxiety-like behavior in behavioral tests of anxiety, such as the elevated plus maze (EPM), open field test, burying defensive test, and light/dark box, as presented in Table 1. In the EPM, ovariectomy decreases the time spent into the open arms, which is considered as higher anxiety and depends on the time post-ovariectomy (Puga-Olguín et al., 2019). Contrarily, anxiolytic drugs like diazepam, hormones like progesterone and estradiol, or natural products like flavonoids, significantly increase the time in the open arms, thereby producing anxiolytic effects (Charoenphandhu et al., 2011; Rodríguez-Landa et al., 2017, 2019). Similar effects are observed in the other behavioral tests for anxiety. Ovariectomy reduces the time spent in the area where anxiogenic stimuli are present, and this time increases when anxiolytic drugs are injected to mice or rats, such as diazepam, estradiol, progesterone, allopregnanolone, or phytoestrogen genistein (Picazo et al., 2006; Llaneza and Frye, 2009; Rodríguez-Landa et al., 2009; Diz-Chaves et al., 2012).

Ovariectomy also increases the incidence of depression-like behavior in some animal models to evaluate despair behavior, including the tail suspension test (TST), forced swim test (FST), and chronic unpredictable stress (CUS). In the TST and FST, ovariectomy increases the time of immobility depending on the time-post ovariectomy (Lagunas et al., 2010; Puga-Olguín et al., 2019), which is considered as an indicator of depression-like behavior. Interestingly, ovariectomized mice and rats treated with antidepressant drugs like maprotiline, or some hormones like progesterone, allopregnanolone, and estradiol, had reduced time of immobility among other behavioral variables (Okada et al., 1997; Tantipongpiradet et al., 2019; Cueto-Escobedo et al., 2020; Khayum et al., 2020; Rodríguez-Landa et al., 2020), which is considered as an antidepressant-like effect. In the particular case of CUS, rats are subjected to several stressors for 4 weeks, which subsequently produces depression-like behavior identified by an increase in the time of immobility in the FST (Lagunas et al., 2010). This effect is dependent on the time post-ovariectomy, which was higher at 20 weeks than at 6 weeks post-ovariectomy. This shows that time post-ovariectomy is an important variable that should be considered in studying the effects of ovariectomy on anxiety- and depression-like behavior, and their potential treatments.

## Concluding Remarks

Studying the effect of the long-term absence of ovarian hormone produced by ovariectomy in mice and rats on anxiety- and depression-like behaviors and the underlying neurochemical and anatomical changes is necessary in understanding neuropsychiatric disorders among women undergoing oophorectomy. Considering that in experimental animals, it is possible to discard the socio-cultural influence that menopause could have on women and its contribution to their emotional and affective disorders (Afridi, 2017; Zhang et al., 2019), the surgical menopause model in mice and rats may provide new evidence for the neurobiological mechanism resulting from ovariectomy (oophorectomy in women) in the long-term. Therefore, this model can be used to explore potential therapeutic strategies to ameliorate emotional and affective symptoms, in addition to physiological, histological, structural, and neuropsychiatric disorders occurring in women subjected to surgical menopause.

As mentioned above, the time elapsed after ovariectomy in rats plays a significant role in the expression of anxiety- and depression-like behavior, and possibly in the effects produced by anxiolytic and antidepressant drugs. Therefore, it is important to consider the time post-ovariectomy when we use such procedure to explore anxiety- and depression-like behavior or potential anxiolytic or antidepressant drugs. Additionally, including control groups of non-ovariectomized rats or referring to the control groups in previously published studies would ensure suitable comparations to progress our research. This would help in enhancing the understanding of behavioral changes associated with surgical menopause and help develop potential pharmacological strategies to ameliorate the negative effects produced by long-term ovariectomy. The model also allows consideration of the post-ovariectomy time frame to achieve better control of the evaluated variables and ensure the reproducibility and comparison of results.

## Author Contributions

JFR-L conceived and wrote this article.

## Funding

This article is part of the SIREI project (DGI: 266502020108) registered by JFR-L, with partial financial support from the Sistema Nacional de Investigadores (SNI, Exp. 32753), CONACyT, Mexico.

## Conflict of Interest

The author declares that the research was conducted in the absence of any commercial or financial relationships that could be construed as a potential conflict of interest.

## Publisher's Note

All claims expressed in this article are solely those of the authors and do not necessarily represent those of their affiliated organizations, or those of the publisher, the editors and the reviewers. Any product that may be evaluated in this article, or claim that may be made by its manufacturer, is not guaranteed or endorsed by the publisher.
